# Ovalitenone Inhibits the Migration of Lung Cancer Cells via the Suppression of AKT/mTOR and Epithelial-to-Mesenchymal Transition

**DOI:** 10.3390/molecules26030638

**Published:** 2021-01-26

**Authors:** Kittipong Sanookpan, Nongyao Nonpanya, Boonchoo Sritularak, Pithi Chanvorachote

**Affiliations:** 1 Cell-Based Drug and Health Product Development Research Unit, Faculty of Pharmaceutical Sciences, Chulalongkorn University, Bangkok 10330, Thailand; 6280112920@student.chula.ac.th (K.S.); nonpanya1988@gmail.com (N.N.); 2 Department of Pharmacology and Physiology, Faculty of Pharmaceutical Sciences, Chulalongkorn University, Bangkok 10330, Thailand; 3 Department of Pharmacognosy and Pharmaceutical Botany, Faculty of Pharmaceutical Sciences, Chulalongkorn University, Bangkok 10330, Thailand; boonchoo.sr@chula.ac.th

**Keywords:** ovalitenone, metastasis, migration, epithelial–mesenchymal transition (EMT), lung cancer

## Abstract

Cancer metastasis is the major cause of about 90% of cancer deaths. As epithelial-to-mesenchymal transition (EMT) is known for potentiating metastasis, this study aimed to elucidate the effect of ovalitenone on the suppression of EMT and metastasis-related behaviors, including cell movement and growth under detached conditions, and cancer stem cells (CSCs), of lung cancer cells. Methods: Cell viability and cell proliferation were determined by 3-[4,5-dimethylthiazol-2-yl]-2,5 diphenyl tetrazo-liumbromide (MTT) and colony formation assays. Cell migration and invasion were analyzed using a wound-healing assay and Boyden chamber assay, respectively. Anchorage-independent cell growth was determined. Cell protrusions (filopodia) were detected by phalloidin-rhodamine staining. Cancer stem cell phenotypes were assessed by spheroid formation. The proteins involved in cell migration and EMT were evaluated by Western blot analysis and immunofluorescence staining. Results: Ovalitenone was used at concentrations of 0–200 μM. While it caused no cytotoxic effects on lung cancer H460 and A549 cells, ovalitenone significantly suppressed anchorage-independent growth, CSC-like phenotypes, colony formation, and the ability of the cancer to migrate and invade cells. The anti-migration activity was confirmed by the reduction of filopodia in the cells treated with ovalitenone. Interestingly, we found that ovalitenone could significantly decrease the levels of N-cadherin, snail, and slug, while it increased E-cadherin, indicating EMT suppression. Additionally, the regulatory signaling of focal adhesion kinase (FAK), ATP-dependent tyrosine kinase (AKT), the mammalian target of rapamycin (mTOR), and cell division cycle 42 (Cdc42) was suppressed by ovalitenone. Conclusions: The results suggest that ovalitenone suppresses EMT via suppression of the AKT/mTOR signaling pathway. In addition, ovalitenone exhibited potential for the suppression of CSC phenotypes. These data reveal the anti-metastasis potential of the compound and support the development of ovalitenone treatment for lung cancer therapy.

## 1. Introduction

Lung cancer is one of the most significant human cancers and continues to be the leading cause of cancer deaths globally [1]. Most cancer deaths are caused by cancer metastasis, accounting for approximately 90% of all cancer deaths. Cancer metastasis is a molecular process that involves the spread of cancer cells from a primary tumor to a different site of the body through the blood and lymphatic vessels [2]. For metastasis to occur, cancer cells must gain their migratory and invasive phenotypes, and they do this through several mechanisms, such as epithelial-to-mesenchymal transition (EMT), whereby cells decrease the apical–basal polarity and switch their pattern and type of adhesion molecules. The EMT process is known for augmenting the cell dissemination potential [3]. EMT is itself a multistep process that results in more mesenchymal phenotypes, including an increase in the expression of N-cadherin, vimentin, snail, and slug, while depleting the adhesive molecules, such as E-cadherin [4]. EMT cells develop filopodia, cellular protrusions from the cell body with focal adhesion, which allows them to form new focal adhesions with the new space in the extracellular matrix (ECM). Then, the cells dissociate from the integrin adherence at the rear side and start cell contraction by the function of actomyosin, thereby causing the movement of the cells [5]. EMT, cell migration, and invasion have been shown to play important roles and have been linked with the high metastasis potential of cancer cells [6]. EMT is not only an essential process in which epithelial cells acquire the motile and invasive phenotypes of mesenchymal cells, but also an importance process for anoikis resistance [7,8].

In lung cancer, ATP-dependent tyrosine kinase (AKT; also known as protein kinase B) is activated, and increased AKT phosphorylation has been found to be associated with cancer metastasis [9]. Studies have shown that the mammalian target of rapamycin (mTOR) also plays an important role in the regulation of cell migration, invasion, and cancer metastasis [10]. Moreover, the activation of focal adhesion kinase (FAK) and its downstream targets, such as the Rho family protein and cell division cycle 42 (Cdc42), are also known to regulate cell migration and the formation of filopodia [11]. There is potential utility in molecularly targeting the components of these signaling pathways for cancer prevention and cancer therapy.

Ovalitenone has been reported to have various pharmacological properties, such as anti-diabetic activity [12], and anti-herpes simplex virus activity [13]; however, to the best of our knowledge, the effect of ovalitenone on metastasis potential and EMT as well as its underlying mechanisms have not yet been reported. Therefore, the objective of this study was to investigate the effect of ovalitenone on cancer migration, invasion, and EMT in lung cancer cells. The results from this study may benefit the development of ovalitenone treatment for anti-metastatic therapy.

## 2. Results

### 2.1. Effect of Ovalitenone on Cell Viability and Proliferation of H460 and A549 Cells

**Figure 1:**
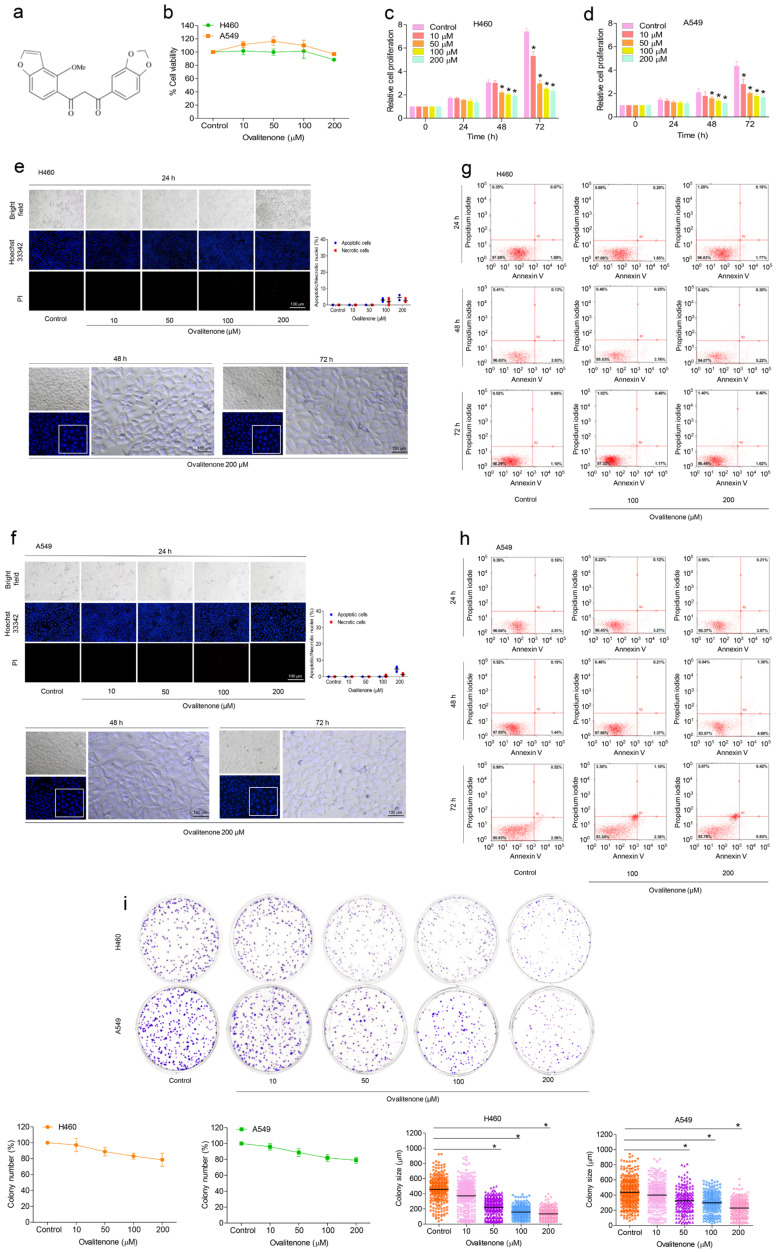
Effect of ovalitenone on cell viability and proliferation of lung cancer H460 and A549 cells. (**a**) Chemical structure of ovalitenone. (**b**) Cell viability of the cells was assessed by 3-[4,5-dimethylthiazol-2-yl]-2,5 diphenyl tetrazo-liumbromide (MTT) assay. (**c**,**d**) Effect of ovalitenone on cell proliferation. (**e**,**f**) The cells were treated with ovalitenone for 24, 48, and 72 h, and apoptotic and necrotic cells were evaluated by Hoechst 33342 and PI staining. (**g**,**h**) Annexin V/PI co-stained cells were examined using flow cytometry. (**i**) Cells were treated as indicated for 7 days, and colony was stained by crystal violet. All data are presented as mean ± SEM (*n* = 3). * *p* < 0.05 compared with untreated cells.

Ovalitenone (Figure 1a), isolated from *Millettia erythrocalyx*, was used in this study. To determine the non-toxic concentrations of ovalitenone to be used in the following experiments, human NSCLC H460 and A549 cells were treated with various concentrations of ovalitenone (0–200 µM) for 24 h, and cell viability was measured by 3-[4,5-dimethylthiazol-2-yl]-2,5 diphenyl tetrazo-liumbromide (MTT) assay. The results reveal that ovalitenone at concentrations lower than 200 µM did not significantly affect the viability in both cells (Figure 1b). For cell proliferation analysis, H460 and A549 cells were treated with various concentrations of ovalitenone (0–200 µM) for 24, 48, and 72 h, and their viability was evaluated. The results show that ovalitenone at concentrations of 0–200 µM had no effect on cell proliferation in H460 and A549 cells at 24 h (Figure 1c,d); however, the rate of proliferation of the treated cells was significantly decreased in a dose-dependent manner at concentrations of 50 to 200 μM at 48 h, while ovalitenone at concentrations of 10 to 200 µM significantly decreased proliferation at 72 h in both cells (Figure 1c,d). In order to investigate the apoptosis induction of ovalitenone in H460 and A549 cells, nuclear staining assay was performed. Cells were treated with ovalitenone at 0–200 µM for 24, 48 and 72 h, and the treated cells were stained with Hoechst 33342 and propidium iodide (PI). We found that ovalitenone at 0–200 μM had no effect on apoptosis or necrosis in H460 and A549 cells (Figure 1e,f). To confirm, apoptosis and necrosis cells in response to ovalitenone treatment were determined by annexin V/PI assay. Flowcytometry analysis revealed that ovalitenone at the concentrations of 100 and 200 µM did not cause either apoptosis or necrosis in both cells at 24, 48, and 72 h (Figure 1g,h). We also confirmed the effect of ovalitenone on cell proliferation by colony formation assay, and the results show that ovalitenone at 50 to 200 µM could decrease the size of the colonies (Figure 1i). Here, non-toxic concentrations of ovalitenone were used for the further anti-migration and invasion experiments described below.

### 2.2. Effect of Ovalitenone on Lung Cancer Cell Migration, Invasion and Filopodia Formation

**Figure 2:**
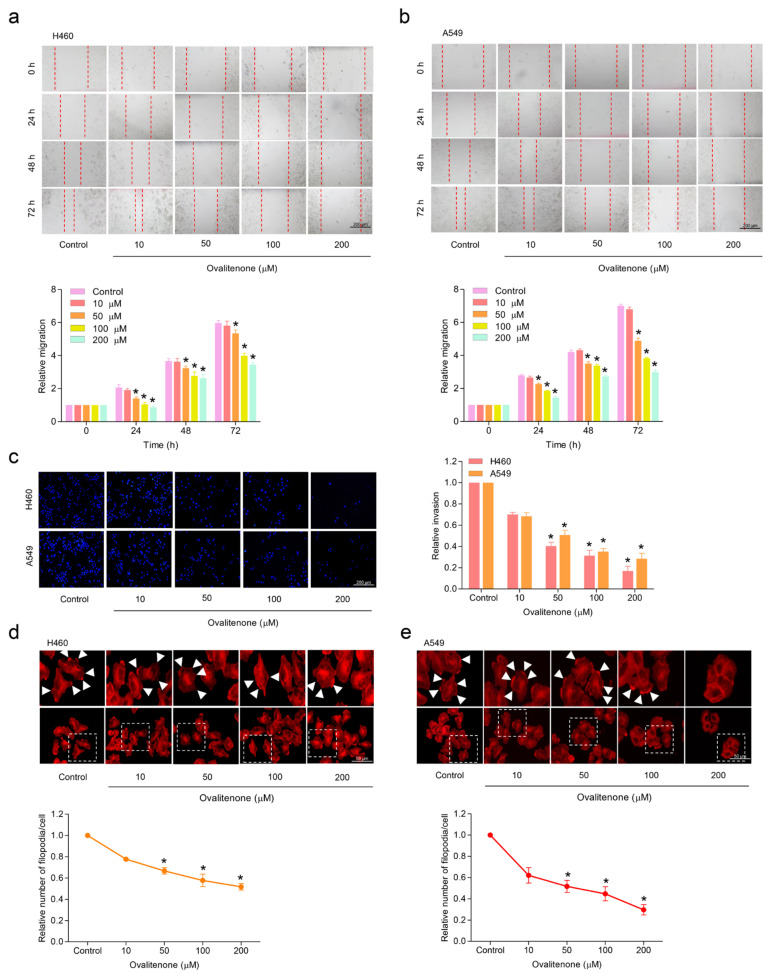
Ovalitenone suppresses cell migration, invasion and filopodia formation. (**a**,**b**) Cells were treated with ovalitenone for 24, 48, and 72 h, and migration activity was determined by wound healing assay. (**c**) Cell invasion was examined by transwell invasion assay. After 24 h, invading cells were stained with Hoechst 33342 and photographed. (**d**,**e**) Cells were treated with ovalitenone for 24 h, filopodia was stained with phalloidin-rhodamine, and the number of filopodia per cells was counted. All data are represented as mean ± SEM (*n* = 3). * *p* < 0.05 compared with untreated cells.

We next investigated the effect of ovalitenone on the migration and invasion properties of lung cancer cells. Cell migration was determined by a wound-healing assay, whereby wounded monolayers of H460 and A549 cells were treated with ovalitenone at non-toxic concentrations (0–200 µM) for 24, 48, and 72 h, respectively. The results reveal that ovalitenone inhibited H460 (Figure 2a) and A549 (Figure 2b) cells’ migration at concentrations of 50 to 200 µM at 24, 48, and 72 h, whereas ovalitenone at 10 µM did not significantly inhibit the cells’ migration (Figure 2a,b). Additionally, cell invasion was determined using a transwell Boyden chamber coated with matrigel. Cells were seeded on the matrigel surface in the presence or absence of ovalitenone (0–200 µM), and the invaded cells at other sites of the membrane were counted at 24 h. Figure 2c shows that the ovalitenone was able to inhibit cell invasion through the matrigel layer. Cell protrusion facilitating cell migration was further evaluated in the cells treated with non-toxic concentrations of ovalitenone. Analysis by phalloidin staining showed that ovalitenone treatment significantly reduced the number of filopodia per cells (Figure 2d,e). Taken together, our results reveal the anti-migratory activities of ovalitenone.

### 2.3. Ovalitenone Attenuates Anchorage-Independent Growth and CSC-Like Phenotypes of Human Lung Cancer H460 and A549 Cells

**Figure 3:**
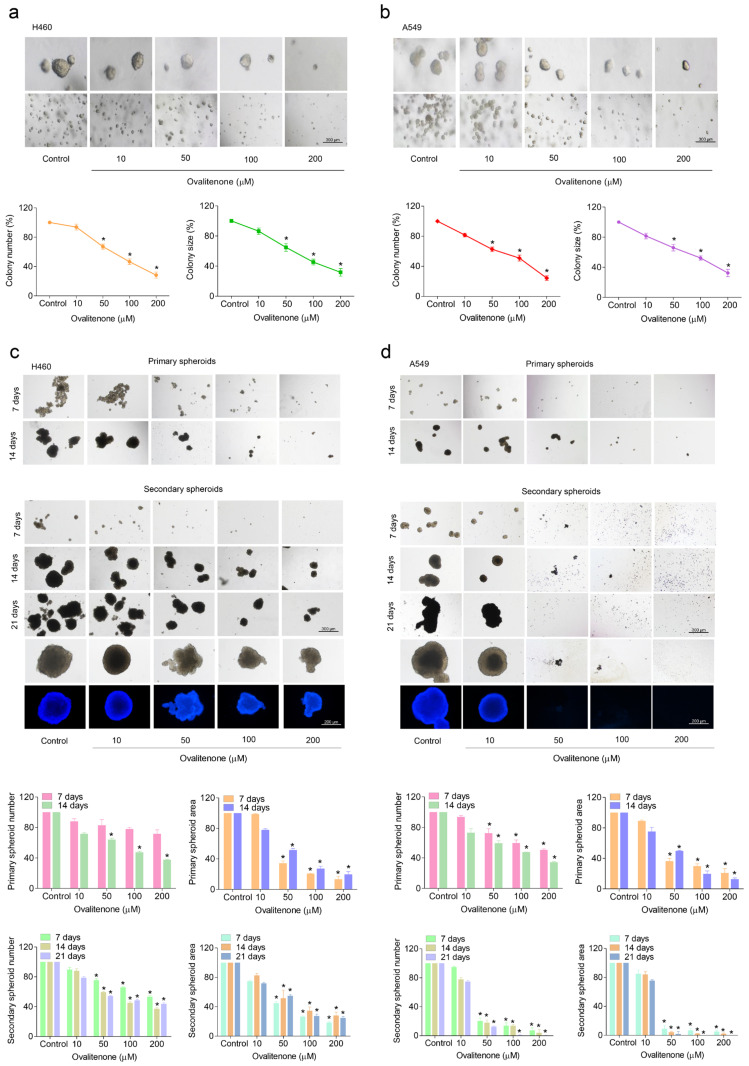
Ovalitenone attenuates anchorage-independent growth and cancer stem cell (CSC)-like phenotypes of human lung cancer H460 and A549 cells. (**a**,**b**) Cells were pre-treated with sub-toxic concentrations of ovalitenone for 24 h, and were subjected to an anchorage-independent growth assay. (**c**,**d**) Cells were pre-treated with non-toxic concentrations of ovalitenone for 24 h, and allowed for 14 days to form primary spheroids. After, the primary spheroids were suspended into single cells to form secondary spheroids for 21 days, the spheroid of cancer stem cell (CSC)-rich population was determined. All data are represented as mean ± SEM (*n* = 3). * *p* < 0.05 compared with untreated cells.

It was previously reported that the process of the anchorage-independent growth of cancer cells reflects anoikis resistance and the metastasis potential of malignant tumor cells [14]. To test whether ovalitenone could suppress such cancer cell growth under attached conditions, H460 and A549 cells were grown in soft agar in the presence or absence of ovalitenone for 14 days. The number and size of the growing cancer colonies were determined and calculated relative to those with the untreated control. The results indicate that the ovalitenone-treated cells (at concentrations of 50 to 200 µM) exhibited a significantly reduced ability to form colonies, and treatment with the compound could attenuate the growth of the cells as indicated by the decrease in colony number and size when compared with the untreated control in both cells (Figure 3a,b). As illustrated in Figure 3a, the colony numbers of H460 cells were significantly reduced by ovalitenone treatment at 50, 100, and 200 μM to 31.05%, 51.48%, and 73.20%, respectively, and the percentages of colony size in response to ovalitenone at the concentrations of 50, 100, and 200 μM were 33.82%, 50.61%, and 63.41%, respectively. The colony numbers of A549 (Figure 3b) cells were significantly reduced by ovalitenone at 50, 100, and 200 μM to 38.93%, 50.37%, and 74.06%, respectively, and the percentages of colony size in response to the compound at 50, 100, and 200 μM were 31.62%, 49.92%, and 66.36%, respectively. These results suggest that ovalitenone at non-toxic doses could inhibit the survival and growth of human lung cancer cells under detached conditions.

As the ability of cancer cells to form spheroids has been used to reflect CSC phenotypes and as CSCs are well known to have a very high metastatic potential, we next studied the effect of ovalitenone on CSCs. H460 and A549 cells were treated with non-toxic concentrations of ovalitenone (0–200 µM) for 24 h, and the cells were subjected to a spheroid formation assay. After secondary spheroids were formed, the results show that the non-treated control cells had a high ability to form tumor spheroids, whereas cells treated with ovalitenone exhibited a reduced presence of tumor spheroids in a dose-dependent manner (Figure 3c,d). Ovalitenone at doses of 50–200 µM significantly decreased the number and size of the primary and secondary spheroids compared with the control in both H460 and A549 cells (Figure 3c,d). Taken together, ovalitenone possessed anti-metastasis activities as it could suppress cancer migration, invasion, and growth under anchorage-independent conditions and could also suppress the CSC-like phenotypes.

### 2.4. Ovalitenone Suppresses EMT via the Suppression of the AKT/mTOR Signaling Pathway

**Figure 4:**
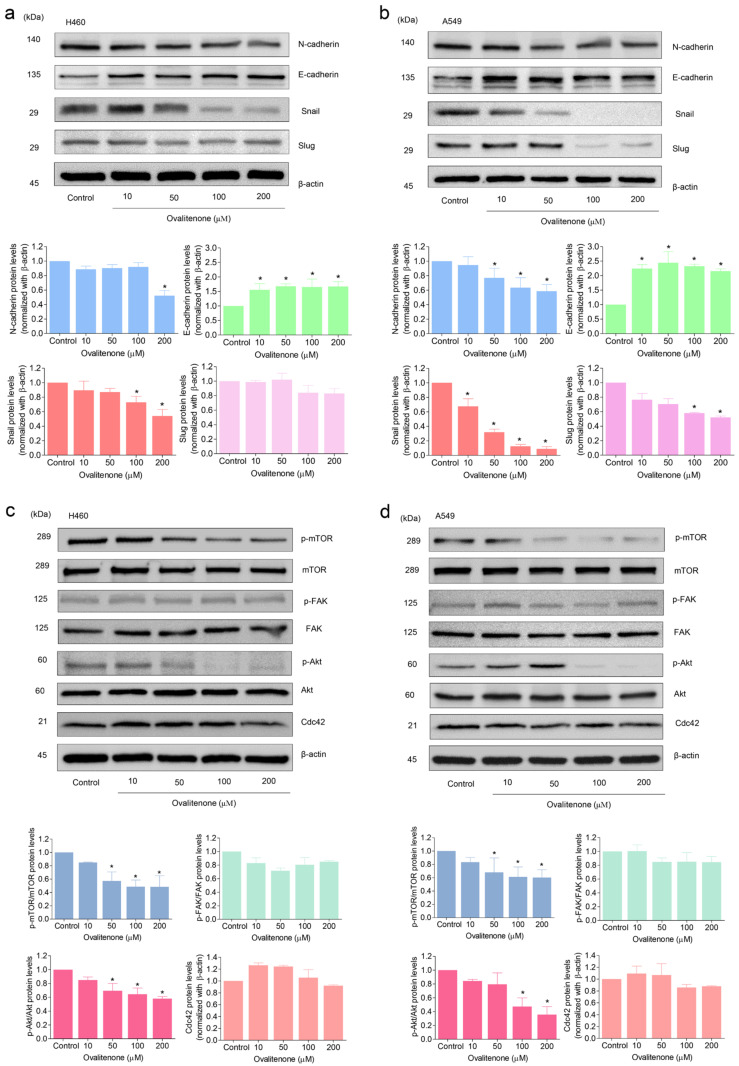
Ovalitenone suppresses epithelial-to-mesenchymal transition (EMT) through inhibition of the ATP-dependent tyrosine kinase (AKT)/mammalian target of rapamycin (mTOR) signaling pathway. (**a**,**b**) After cells treated with non-toxic concentrations of ovalitenone for 24 h, the expression levels of N-cadherin, Snail and Slug were determined by Western blotting. (**c**,**d**) Levels of phosphorylated mTOR (Ser2448), phosphorylated focal adhesion kinase (FAK) (Tyr397), phosphorylated Akt (Ser473), and Cdc42 were determined by Western blot analysis. All data are represented by mean ± SEM (*n* = 3). * *p* < 0.05 compared with untreated cells.

EMT has been well linked with metastasis and is known to be a cellular process that can facilitate migration and invasion, anoikis resistance, and the CSCs of cancer cells [15]. In evaluating the underlying mechanism of ovalitenone action, we first determined the EMT makers in cells exposed to ovalitenone. Cells were treated with ovalitenone (0–200 µM) for 24 h, and the levels of the well-recognized EMT markers, namely, N-cadherin, E-cadherin, snail, and slug, were determined. The results indicate that ovalitenone significantly decreased the cellular levels of N-cadherin and snail, as well as increased E-cadherin in a dose-dependent manner in H460 and A549 cells (Figure 4a,b).

Moreover, the upstream regulatory cell signals of EMT and the controllers of cell migration, such as FAK, AKT, mTOR, and Cdc42, were further analyzed. Cdc42 has been implicated as playing an important role in the formation of filopodia, and the level of the protein has also been linked with increased migration [16]. The results reveal that ovalitenone was able to decrease the active form of mTOR, i.e., p-mTOR (phosphorylated at Ser2448), in H460 and A549 cells at 50–200 µM (*p* < 0.05). Likewise, the active form of AKT, i.e., p-AKT (phosphorylated AKT at Ser473), was found to be significantly reduced in response to 50–200 µM ovalitenone in H460 cells (Figure 4c); however, we found a significant reduction of the p-AKT protein only at concentrations of 100 and 200 µM in A549 cells (Figure 4d). Further, Cdc42 and p-FAK (phosphorylated at Tyr397) were not affected by the treatment in both lung cancer cells (Figure 4c,d).

**Figure 5:**
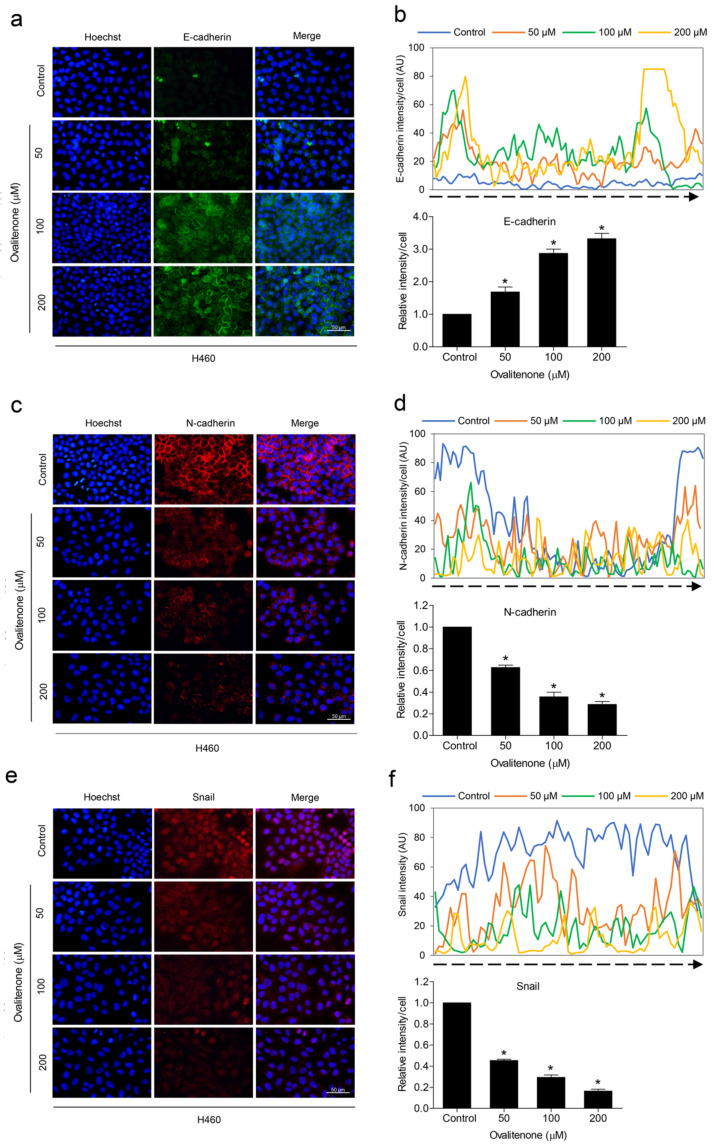
Ovalitenone suppresses EMT. (**a**,**c**,**e**) H460 cells were treated with non-toxic concentrations of ovalitenone for 24 h. The cells were co-stained with anti-E-cadherin, N-cadherin, Snail antibodies, and Hoechst 33342. The expression of E-cadherin, N-cadherin, and Snail was examined using immunofluorescence. (**b**,**d**,**f**) The fluorescence intensity was analyzed by ImageJ software. Values represent the mean ± SEM. (*n* = 3). * *p* < 0.05 compared with untreated cells.

**Figure 6:**
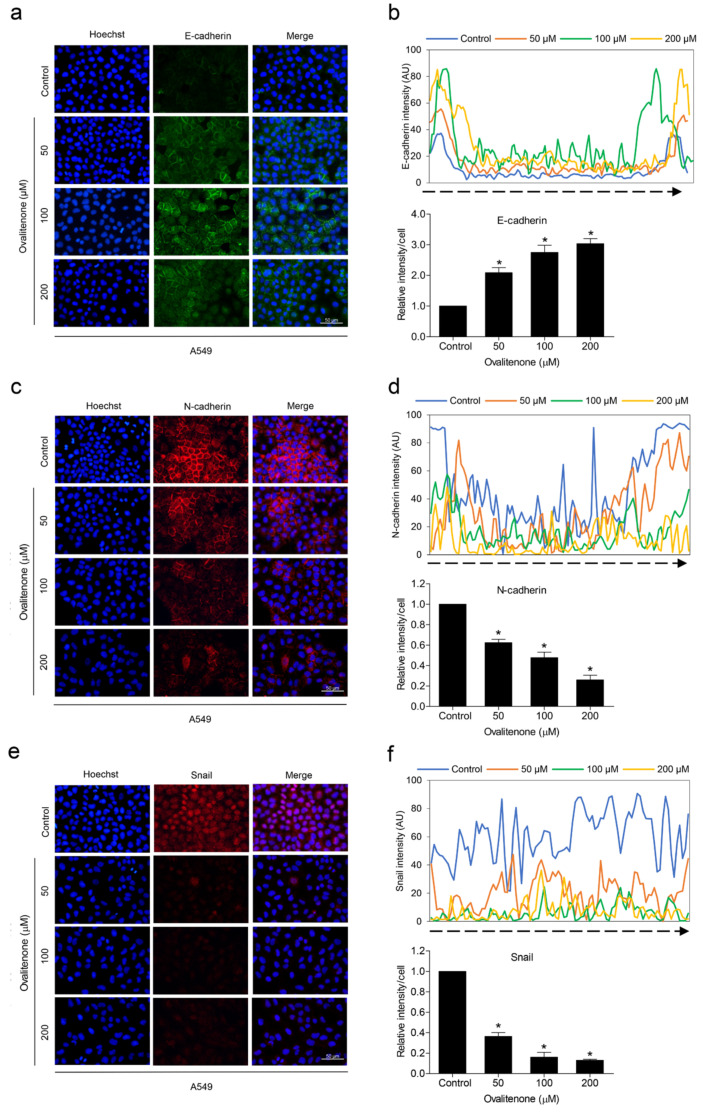
Ovalitenone suppresses EMT. (**a**,**c**,**e**) A549 cells were treated with ovalitenone at indicated concentrations for 24 h. The cellular levels of E-cadherin, N-cadherin, and Snail were determined by immunofluorescence analysis. (**b**,**d**,**f**) The fluorescence intensity was analyzed by ImageJ software. Values represent the mean ± SEM. (*n* = 3). * *p* < 0.05 compared with untreated cells.

We further confirmed the inhibitory effect of ovalitenone on E-cadherin, N-cadherin, and snail by immunofluorescence staining. Figure 5 shows that ovalitenone significantly decreased the levels of N-cadherin and snail, whereas it upregulated E-cadherin in H460 cells. Consistently, similar results from the immunofluorescence analysis in A549 cells were found, as shown in Figure 6. These results indicate that ovalitenone suppressed EMT as well as inhibited lung cancer cell motility through inhibition of the AKT/mTOR signaling pathway.

## 3. Discussion

Human lung cancer has been shown to be the leading cause of cancer-related deaths worldwide [1]. Cancer cell migration is one of the cancer hallmarks and is critical for the success of metastasis [17]. Currently, the 5-year survival of lung cancer with metastasis is considered very low (about 18%) and most lung cancer patients present with metastatic disease at the first diagnosis [18]. As metastasis has been shown to be an important factor of cancer death in lung cancer, approaches that attenuate the metastasis potential are of interest as promising means to improve the clinical outcome. In fact, cancer metastasis is a complex process involving the spread of cancer cells from a primary tumor to different sites of the body through the blood and lymphatic vessels [2]. During the process of metastasis, cancer cells must gain the ability to migrate and invade tissue, resist detachment-induced apoptosis (anoikis), survive in the circulatory systems, and establish new colonies at metastatic sites [19].

EMT, a switching process from the epithelial phenotype of an adherent cell to a motile mesenchymal phenotype is known to facilitate cancer metastasis in several human cancers [20]. Besides, EMT has been highlighted as an important target of anti-cancer drugs as well as a key treatment strategy [21]. Previously, research has shown that natural product-derived compounds exhibit potential activities in the inhibition of cancer cell migration and invasion, as well as EMT. For instance, gigantol, a bibenzyl compound extracted from *Dendrobium draconis*, has been shown to suppress EMT in non-small cell lung cancer H460 cells [22]. Petpiroon et al. reported that phoyunnanin E inhibited the motility of lung cancer cells via the suppression of EMT and cancer metastasis-related integrins, such as integrin αv and integrin β3 [23]. Our previous results showed that ephemeranthol A, a natural compound isolated from *D. infundibulum*, also exhibited inhibitory effects on migration and EMT via suppression of the FAK-AKT signaling pathway [24]. Similarly, batatasin III was reported to have an inhibitory effect on cancer migration and invasion by suppressing EMT [25].

Cell migration results from a highly integrated multistep process that is regulated by various signaling molecules, such as Rho-GTPases, and focal adhesion kinase (FAK) [26,27]. Focal adhesion kinase (FAK) is an intracellular protein-tyrosine kinase (PTK) involving in the regulation of cell cycle progression, survival, and migration. The ability of cancer cells to migrate has been found to be linked to increased FAK expression, phosphorylation, and catalytic activity [28]. The phosphorylation of Y397 of FAK has been reported to play a crucial role in FAK-mediated cell migration [29]. Studies have shown the potential anti-migration benefit of FAK/AKT inhibition. Plaibua et al. reported that artonin E inhibited cell migration and invasion via a FAK-AKT-dependent mechanism [30]. Cardamonin has also been shown to inhibit cancer metastasis by suppressing the PI3K/AKT/mTOR pathway [31]. Focusing on AKT, it was shown that AKT and its related cellular pathways were highly activated in migrating cells [32]. In general, AKT is known to be a central signaling pathway for several cell behaviors, such as proliferation, survival, and motility [33]. Studies have shown that the activated AKT plays an important role in cancer cell metastasis and the EMT process [3,34].

AKT can activate mTOR through the direct phosphorylation of tuberous sclerosis complex 2 (TSC2) [35]. mTOR is a molecule downstream of the AKT pathway that has been shown to regulate a series of critical cellular metabolism, migration, invasion, and angiogenesis [36]. We assessed the expression of proteins and found that ovalitenone could inhibit the active AKT and mTOR, which could at least in part be responsible for the anti-migration activity of the compound. The regulatory link between AKT/mTOR and EMT has been revealed in several studies. One study showed that transforming growth factor beta (TGF-β) induced EMT and cell invasion via the activation of mTORC2 kinase activity [37]. Moreover, MTORC1 and mTORC2 were shown to be modulators of EMT in cancer, while the silencing mTORC1 and mTORC2 resulted in an increase in E-cadherin and decrease in N-cadherin, vimentin, and snail, and other characteristics of mesenchymal to epithelial transition (MET) [38].

Recently, CSCs, a small groups of cancer cells within tumors having stem cell properties, were shown to be implicated in the initiation, progression, metastasis, and recurrence of lung cancer [39]. The characteristics of CSCs include a renewal ability, differentiation, high invasiveness, and resistance to chemotherapy. CSCs are the main factor contributing to the current low rate of therapeutic success [40]. According to the data, CSCs are considered to be the critical driver of cancer aggressiveness, including high tumor maintenance, the probability of metastasis, avoidance of the immune system, resistance to chemotherapy, and cancer relapse [41]. Interestingly, our results suggest a potential role of ovalitenone in the inhibition of CSCs, as the treatment of lung cancer cells at non-toxic concentrations could significantly decrease the ability to form tumor spheroids (Figure 3c,d). The ability of a single cancer cell to generate a growing tumor spheroid has been used for CSC assessment in many research studies [42,43]. Besides, the regulatory pathways that are widely accepted to regulate and maintain CSCs in cancers, including the AKT and mTOR signals, were suppressed by the treatment of ovalitenone (Figure 4c,d). AKT and mTOR were demonstrated to be critical cell signaling pathways for CSC maintenance in many cancers, including lung cancer [44]. Moreover, strategies that target these pathways have been shown to be promising approaches for lung cancer treatment [45]. EMT and CSCs were shown to be involved in a close crosstalk network. In some studies, EMT was shown to increase CSC phenotypes in cancers [46,47]. Interestingly, EMT transcription factors, including snail, twist, and ZEB families, are also known to confer multiple CSC properties [48].

The lung cancer cells H460 and A549 cells were used as cell models in the present study. The H460 and A549 cells have Kras mutations, which was shown to associate with metastatic potentials [49,50]. In lung cancer, Ras mutations are found in around 32%, and Kras is the most common member of the mutated family [51]. Kras protein, a small guanine triphosphatase (GTPase), functions in the transducing signal of receptor epidermal growth factor receptor (EGFR) and tyrosine kinases. Moreover, Ras proteins recruit and activate downstream effectors, such as AKT and ERK pathways that in turn affect cell growth, differentiation and survival [52]. As we focus on the EMT process, Kras is known to activate EMT, resulting in tumor progression and metastasis. Kras mutations were shown to induce EMT through several mechanisms including the upregulation of the EMT transcription factors, snail and slug [53]. In terms of cell migration, several studies have utilized both H460 and A549 cell lines for investigating migratory activity and anti-migratory investigation. For instance, Xia Rongmu et al. demonstrated the anti-migration and anti-invasion of hesperidin in A549 cells [54]. Lv Xiao-qin et al. used H460 and A549 cells as models for studying the inhibitory effects of Honokiol on EMT-mediated motility and migration [55]. Furthermore, Ophiopogonin B was shown to suppresses metastasis-related activities of A549 cells [56].

Recently, natural compounds from plants have been receiving increasing attention either as the potential drugs or lead compounds in drug discovery [57]. *Millettia erythrocalyx*, which is widely distributed in the tropical and subtropical regions of the world, such as China and Thailand [58], is one natural species that has attracted interest and is a source of ovalitenone. Ovalitenone from *Millettia erythrocalyx* has been used in various traditional medicines, such as for treating bleeding piles, fistulous sores, bronchitis, and coughs [59,60]. Here, we found that ovalitenone inhibited cell migration and invasion, as well as EMT in human lung cancer H460 and A549 cells. Ovalitenone suppressed the formation of cancer cell colonies and CSC spheroids. Moreover, the treatment of human lung cancer cells with ovalitenone at non-toxic concentrations significantly decreased the levels of N-cadherin, snail, and slug, while it increased E-cadherin (Figure 4a,b), indicating the effect of this compound in terms of its inhibition of EMT; thereby, resulting in the inhibition of cell movement from the suppression of the AKT/mTOR pathways (Figure 4c,d).

## 4. Materials and Methods

### 4.1. Isolation of Ovalitenone

Ovalitenone was isolated from the roots of *Millettia erythrocalyx* Gapnep [60]. Briefly, the dried and powdered roots of this plant (8 kg) were extracted with n-hexane, CHCl_3_, and MeOH at room temperature to afford n-hexane extract (91 g), CHCl_3_ ex-tract (87 g), and MeOH extract (429 g) after removal of the solvent. This material was subjected to vacuum-liquid chromatography on silica gel (ethyl acetate-pet. ether gradient) to give A-E fractions. Fraction A3 (169 mg) was fractionated by column chromatography over silica gel (toluene), giving ovalitenone (26 mg; R_f_ 0.24, silica gel, CHCl_3_-toluene 1:2). Its purity was determined using NMR spectroscopy. Ovalitenone with more than 95% purity was used in this study. The chemical structure of ovalitenone is shown in Figure 1a. Ovalitenone was dissolved in dimethyl sulfoxide (DMSO) (Sigma Chemical, St. Louis, MO, USA). The stock solution was diluted with culture medium to achieve the desired concentrations, containing less than 0.1% DMSO at final concentrations.

### 4.2. Cell Culture and Chemicals

Human NSCLC H460 and A549 cells were obtained from the American Type Culture Collection (Manassas, VA, USA). H460 and A549 cells were cultured in Roswell Park Memorial Institute (RPMI) 1640 medium (Gibco, Grand Island, NY, USA) and Dulbecco’s Modified Eagle’s Medium (DMEM) (Gibco, Grand Island, NY, USA), respectively. The culture media were supplemented with 10% fetal bovine serum (FBS) (Merck, DA, Germany), 2 mM L-glutamine (Gibco, Grand Island, NY, USA) and 100 U/mL penicillin and streptomycin (Gibco, Grand Island, NY, USA), and incubated at 37 °C with 5% CO_2_ in a humidified incubator. 3-[4, 5-dimethylthiazol-2-yl]-2,5-diphenyltetrazolium bromide (MTT), Propidium iodide (PI), Hoechst 33342, Triton X-100 and phalloidin-rhodamine were obtained from Sigma Chemical, Inc. (St. Louis, MO, USA). Bovine serum albumin (BSA), and glycerol were obtained from Merck (DA, Germany). Fetal bovine serum (FBS), and agarose were obtained from Bio-Rad Laboratories (Hercules, CA, USA). Antibodies for N-cadherin (#13116), E-cadherin (#3195), Slug (#9585), Snail (#3879), p-mTOR (#5536), mTOR (#2983), p-FAK (#3283), FAK (#3285), p-AKT (#4060), AKT (#9272), Cdc42 (#2466), β-actin (#4970), as well as peroxidase-conjugated secondary antibodies were obtained from Cell Signaling Technology, Inc. (Danvers, MA, USA). Radioimmunoprecipitation assay (RIPA) buffer was obtained from Cell Signaling Technology, Inc. (Danvers, MA, USA).

### 4.3. Cell Viability and Cell Proliferation Assay

Cell viability and cell proliferation were examined by MTT assay. Cells (1×10^4^ cells/well for cell viability and 2×10^3^ cells/well for cell proliferation) were seeded onto each well of a 96-well plate and incubated overnight for cell attachment. Cells were treated with various concentrations of ovalitenone (0–200 µM) for 24 h for the cell viability assay, and the time was extended to 48 and 72 h for cell proliferation assay. After treatments, the culture media were removed and incubated with 400 μg/mL of MTT at 37 °C for 3 h and the formazan crystals were solubilized in 100 μL DMSO and measured at wavelength 570 nm with microplate reader (Anthros, Durham, NC, USA). The percentage of cell viability was calculated as absorbance of ovalitenone-treated cells relative to the untreated cells. The proliferation rates were determined using the following equation: OD at indicated time/OD at time 0.

### 4.4. Nuclear Staining Assay

H460 and A549 cells were seeded onto 96-well plates at a density of 1×10^4^ cells/well and incubated at 37 °C with 5% CO_2_ for overnight. Cells were treated with ovalitenone at various concentration (0–200 µM) and incubated at 37 °C with 5% CO_2_ for 24 h. After treatments, cells were co-stained with 10 µM of Hoechst 33342 (Sigma, St. Louis, MO, USA) and propidium iodide (PI) (Sigma, St. Louis, MO, USA) for 15 min at 37 °C. Cells were visualized and imaged using a fluorescence microscopy (Olympus DP70, Melville, NY, USA). Three random fields were captured at 20 × magnification and then the percentages of apoptotic cells were calculated.

### 4.5. Apoptosis Assay

Apoptotic and necrotic were evaluated using Annexin V-FITC apoptosis Kit (Thermo Fisher Sciencetific, Waltham, MA, USA). Cells were treated with ovalitenone at concentrations 100 and 200 µM, and incubated at 37 °C for 24, 48 and 72 h. Cells were suspended with 100 µL of 1 × binding buffer and incubated in 5 and 1 µL of PI in the dark at room temperature for 15 min. Binging buffer added 400 µL of incubation buffer, and cells were analyzed using guava easyCyteTM flow cytometry (Merk, DA, Germany).

### 4.6. Colony Formation Assay

H460 and A549 cells were seeded onto 6-well plates at a density 300 cells/well and incubated at 37 °C for overnight. The culture medium was replaced with fresh medium containing ovalitenone at non-toxic concentrations (0–200 µM), and the cells were incubated for 7 days. The cells were washed twice with PBS, fixed with 4% paraformaldehyde (Sigma Chemical, St. Louis, MO, USA) for 20 min, and 30 min staining with crystal violet solution at room temperature. After washing with tap water, the colonies were counted, and the results were representative of three independents.

### 4.7. Migration Assay

Migration was determined by wound healing assay. H460 and A549 cells were cultured as monolayer in 96-well plates. The bottom of each well was scratched using sterile 1-mm-wide pipette tip (P200 micropipette tip). Culture medium was removed and washed by 1 × PBS. The monolayer cells were incubated with ovalitenone at non-toxic concentrations (0–200 µM) at 37 °C for 24, 48 and 72 h, respectively. At indicated time points, migration was observed under a phase contrast microscope (Olympus, Melville, NY, USA) and images were captured using an Olympus DP70 digital camera with Olympus DP controller software (Olympus). The wound area was determined by ImageJ software, and the percentage of wound closure was calculated using the following equation:

(1) $$wound\;closure\left(\%\right)\;=\left[\frac{\left(A_{0}-A_{\Delta h}\right)}{A_{0}}\right]\times 100\;$$

where *A*_0_ is the area of the wound measured immediately after scratching (0 h), and *A_∆h_* is the area of the wound measured at 24, 48 or 72 h after treatment. Relative cell migration was calculated by dividing the percentage change in the wound space of treated cells by that of the control cells in each experiment.

### 4.8. Cell Morphology and Filopodia Characterization

Cell morphology and filopodia was investigated by a phalloidin-rhodamine staining assay. The cells were seeded in 96-well plates for overnight. Cells were treated with ovalitenone at non-toxic concentration (0–200 µM) for 24 h. After treatment, the treated cells were washed with 1 × PBS and fixed with 4% paraformaldehyde (Sigma Chemical, St. Louis, MO, USA) in 1 × PBS for 10 min at 37 °C. Then, cells were permeabilized with 0.1% Triton X-100 (Sigma Chemical, St. Louis, MO, USA) in 1 × PBS for 4 min and blocked with 0.2% bovine serum albumin (BSA) (Merck, DA, Germany) for 30 min. Subsequently, cells were incubated with a phalloidin-rhodamine (Sigma Chemical, St. Louis, MO, USA) in 1 × PBS for 15 min, and mounted with 50% glycerol (Merck, DA, Germany). Cell morphology and filopodia were assessed under fluorescence microscope (Nikon ECLIPSE Ts2, Tokyo, Japan). The relative number of filopodia/cells was determined as the number of filopodia/cells of the ovalitenone-treated cells divided by the number of control cells.

### 4.9. Invasion Assay

Cell invasion was performed by using transwell Boyden chamber (8 μm pore size; BD Bioscience, MA, USA). H460 and A549 cells were treated with ovalitenone at non-toxic concentrations (0–200 µM) for 24 h before subjecting to invasion evaluation. The upper chamber was coated with 0.5% Matrigel (BD Biosciences, San Jose, CA, USA) overnight at 37 °C. Then, the treated cells were seeded at a density of 2 × 10^4^ cells/well in the upper chamber supplemented with serum-free medium, while the complete medium containing 10% FBS (Merck, DA, Germany) was added to the lower chamber compartment as a chemoattractant. After incubation at 37 °C for 24 h, the non-invading cells in the upper chamber were removed with a cotton swab and the invading cells in the lower chamber were fixed with cold methanol (Merck, DA, Germany) for 10 min and stained with 10 μM of Hoechst 33342 (Sigma, St. Louis, MO, USA) for 10 min. Finally, the stained cells were visualized and captured using a fluorescence microscope (Nikon ECLIPSE Ts2, Tokyo, Japan). Relative invasion was calculated by dividing the number of invaded cells treated with ovalitenone compared to the non-treated cells in each experiment.

### 4.10. Anchorage-Independent Growth Assay

Anchorage-independent growth was determined by the soft agar colony-formation assay. Briefly, H460 and A549 cells were pre-treated with ovalitenone at non-toxic concentrations (0–200 µM) for 24 h at 37 °C. The bottom layer was prepared by using a 1:1 mixture of RPMI-1640 or DMEM medium containing 10% FBS (Merck, DA, Germany) and 1% agarose; then, it was allowed to solidify for 20 min at 4 °C. The upper layer consisting of 0.3% agarose gel with 10% FBS (Merck, DA, Germany) and containing 1x10^3^ cells/mL was prepared and added. RPMI-1640 or DMEM medium containing 10% FBS (Merck, DA, Germany) was added over the upper layer. Cells were incubated for 14 days at 37 °C and colony formation were counted and captured using a phase-contrast microscope (Nikon ECLIPSE Ts2, Tokyo, Japan). Relative colony number and size was counted and calculated by dividing the values of the treated cells by the non-treated cells.

### 4.11. Spheroid Formation Assay

H460 and A549 cells were pre-treated with non-toxic concentrations of ovalitenone (0–200 µM) for 24 h in an environment of 37 °C with 5% CO_2_. The treated cells were seeded at a density of 2.5 × 10^3^ cells/well onto an ultralow-attachment plate in culture medium containing 1% (*v/v*) FBS (Merck, DA, Germany) for 14 days to form primary spheroids. The primary spheroids were suspended into single cells, and then these cells were grown in a 24-well ultralow-attachment plate at a density of 2.5 × 10^3^ cells/well for 21 days to form secondary spheroids. At Days 14 and 21, the numbers and sizes of secondary spheroids were determined and imaged using a phase-contrast microscopy (Nikon ECLIPSE Ts2, Tokyo, Japan).

### 4.12. Western Blot Analysis

After ovalitenone at non-toxic concentrations (0-200 µM) treatment, cells were incubated on ice for 40 min with RIPA buffer, 1% Triton X-100, 100 mM PMSF, and a protease inhibitor. Cell lysates were analyzed for protein content using the BCA protein assay kit from Pierce Biotechnology (Rockford, IL). Equal amounts of protein samples (approximately 60–100 µg) were separated by sodium dodecyl sulfate polyacrylamide gel electrophoresis (SDS-PAGE) and transferred to polyvinylidene difluoride (PVDF) (Bio-Rad Laboratories Inc., CA, USA) or nitrocellulose membranes (Bio-Rad Laboratories Inc., CA, USA). The resulting blots were blocked for 2 h with 5% (*w/v*) non-fat dry milk (Merck, DA, Germany) in TBS-T (Tris-buffer saline with 0.1% Tween containing 25 mM Tris-HCl (pH 7.5), 125 mM NaCl, and 0.1% Tween 20) and incubated with the specific primary antibodies against N-cadherin, E-cadherin, Snail, Slug, p-mTOR, mTOR, p-FAK, FAK, p-AKT, AKT, Cdc42, and β-actin (Cell Signaling, Danvers, MA, USA) at 4 °C overnight. After three washed with TBS-T, the membranes were incubated with horseradish peroxidase (HRP)-conjugated secondary antibodies (Cell Signaling, Danvers, MA, USA) for 2 h at room temperature. Finally, the immune blots were detected by enhanced chemiluminescence (Supersignal West Pico; Pierce, Rockford, IL, USA) and quantified using ImageJ software (NIH, Bethesda, MD, USA).

### 4.13. Immunofluorescence Assay

Cells were seeded onto 96-well plates at a density 1 × 10^4^ cells/well and incubated overnight. The cells were treated with ovalitenone for 24 h. The cells were washed twice with 1 × PBS and fixed with 4% paraformaldehyde for 20 min, permeabilized with 0.1% Triton-x in PBS for 20 min, and blocked with 4% BSA in 1 × PBS for 30 min at room temperature. The cells were incubated with primary antibodies at 4 °C overnight, the cells were washed twice with 1 × PBS and incubated with secondary antibodies for 1 h in the dark at room temperature. The cells were washed with 1 × PBS and incubated with Hoechst 33342 (Sigma, St. Louis, MO, USA) for 20 min in the dark, rinsed with 1 × PBS and mounted by 50% glycerol (Merck, DA, Germany). Confocal images were assessed under fluorescence microscope with a 40 × objective lens (Nikon ECLIPSE Ts2, Tokyo, Japan) and the analysis was assessed by ImageJ software.

### 4.14. Statistical Analysis

Statistical differences between multiple groups were analyzed using an analysis of variance (ANOVA). The data were presented as the mean ± standard error of the mean (SEM) of three independent experiments with three replicates per experiment. All *p*-values of less than 0.05 (*) were considered as statistically significant.

## 5. Conclusions

**Figure 7:**
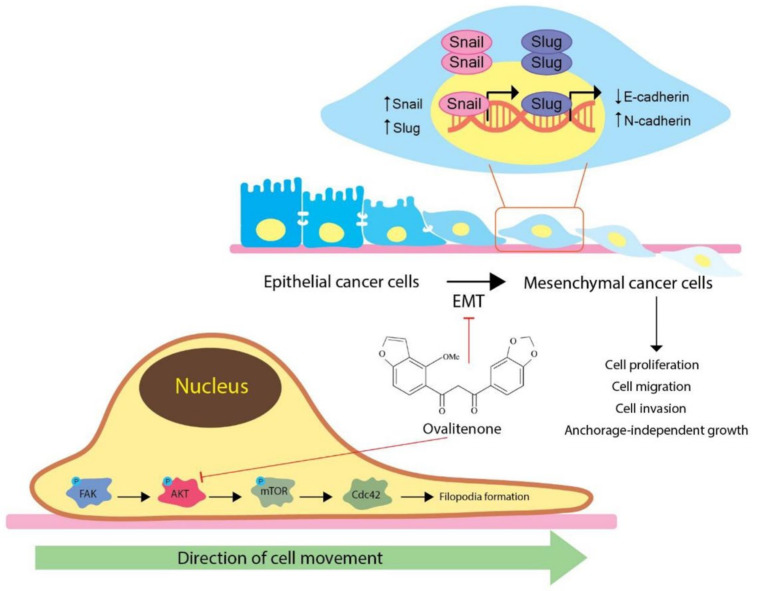
The schematic diagram summarizes the underlying mechanism of ovalitenone-attenuating EMT in lung cancer.

In conclusion, we showed here for the first time that ovalitenone inhibits cancer migration and invasion, as well as growth in an anchorage-independent manner, and we also provided the detailed mechanism of action. The results indicate that ovalitenone suppressed EMT and AKT/mTOR signals, which are important controllers of cancer cell migration, invasion, and metastasis (Figure 7). Additionally, ovalitenone could potentially inhibit the formation of CSC-like phenotypes in lung cancer cells. These data could support the development of ovalitenone treatment for anti-metastasis therapy.

## Data Availability

Data is contained within the article.

## Sample Availability

Samples of the compounds are not available from the authors.
